# The Synergistic Effects of Heat Shock Protein 70 and Ginsenoside Rg1 against Tert-Butyl Hydroperoxide Damage Model In Vitro

**DOI:** 10.1155/2015/437127

**Published:** 2015-01-15

**Authors:** Dan Lu, Anding Xu, Hongcheng Mai, Jiayi Zhao, Chanjuan Zhang, Renbin Qi, Huadong Wang, Daxiang Lu, Lihong Zhu

**Affiliations:** ^1^Department of Pathophysiology, Institute of Brain Research, Key Laboratory of State Administration of Traditional Chinese Medicine of the People's Republic of China, School of Medicine, Jinan University, Guangzhou, Guangdong 510632, China; ^2^Department of Internal Neurology, Guangzhou Overseas Chinese Hospital, The First Affiliated Hospital of Jinan University, Guangzhou, Guangdong 510632, China

## Abstract

Neural stem cells (NSCs) transplanted is one of the hottest research to treat Alzheimer's disease (AD), but cholinergic neurons from stem cells were also susceptible to cell death which Heat shock protein 70 (HSP70) was affirmed to reverse. Related to cognitive impairment, cholinergic nervous cells should be investigated and ginsenoside Rg1 (G-Rg1) was considered to increase them. We chose tert-butyl hydroperoxide (t-BHP) damage model to study in vitro. Functional properties of our recombination plasmid pEGFP-C2-HSP70 were affirmed by SH-SY5Y cells. To opposite the transitory appearance of HSP70, NSCs used as the vectors of HSP70 gene overexpressed HSP70 for at least 7 days in vitro. After transfection for 3 days, G-Rg1 pretreatment for 4 hours, and coculture for 3 days, the expression of acetylcholinesterase (ChAT), synaptophysin, and the ratio of NeuN and GFAP were assessed by western blot; Morphological properties were detected by 3D reconstruction and immunofluorescence. ChAT was markedly improved in the groups contained G-Rg1. In coculture system, the ratio of neurons/astrocytes and the filaments of neurons were increased; apoptosis cells were decreased, compared to monotherapy (*P* < 0.05). In conclusion, we demonstrated that, as a safe cotreatment affirmed in vitro, overexpression of HSP70 in NSCs plus G-Rg1 promoted nervous cells regeneration from chronic oxidative damage.

## 1. Introduction

AD is the most common form of dementia [1], which lack of efficiency therapy; But NSCs is currently one of the hottest research fields to treat AD in biology, which the traits of self-renewal and multipotent, differentiation and integration of transplanted NSCs into mature neuronal networks. AD patients present a large loss of basal forebrain cholinergic nervous cells (neurons and astrocytes), as well as a decrease of ChAT activity and cholinergic input, which associated with the cognitive deficits [2]. Some studies manifested that NSCs transplantation may replenish the supply for extensive loss of neurons [3, 4], but no remarkable directional differentiation [5].

G-Rg1, a steroidal saponin of high abundance in ginseng, appeared to be more potent than other major active compounds of ginseng in improving acquisition impairment [6]. G-Rg1 has been demonstrated to promote the neural phenotype differentiation and cell proliferation of human adipose-derived stem cells [7] and to ameliorate memory deficiency by increasing ACh levels in the hippocampus. Separately, ChAT NSCs improved both the physical activity and cognitive function of aging animals [8]. It is worth noting that the proliferation and differentiation of transplanted NSCs might be not significantly influenced [9], while the nervous cells differentiated from NSCs had to face still accusation which had demonstrated that the neurotoxic properties of A*β* impaired them by oxidative mechanisms [10]. So the crucial problem of the tropism of NSCs transplantation in AD is to control the differential NSCs against the still accusation. Although gene therapy has significant clinical relevance, it has been proven that using gene transfer for HSP70 overexpression improved neuronal survival [11, 12], which may lead to a promising approach for treating AD by inhibition of oxidative stress [13], upregulation of the expression of A*β*-degrading enzyme [12, 14], antiapoptosis via inhibition of both Caspase-dependent and Caspase-independent programmed cell death pathways [15], and protection from A*β*-induced synaptic and neurites damage [16, 17].

As a key player in AD pathogenesis, the exact mechanism by which A*β* damages nervous cells is unclear [18], including generation of oxidant species [19], inhibition of glucose uptake [20], and deprivation of glia support [21]. So monotherapy for a single etiology might not modify the disease process. Hence, in this study, we attempted the cotreatment by combining overexpression of HSP70 in NSCs with G-Rg1 to protect nervous cells after* t*-BHP (an oxidative agent which mimics oxidative cell injury by A*β* for pilot study) insult [22]. There are three valuable indexes in vitro: (1) the expression of ChAT, (2) the production of neurons levels, the growth of neurites, and the formation of synapses [23, 24], and (3) the rate of nervous cells survival.

## 2. Materials and Methods 

### 2.1. Materials

The SH-SY5Y human neuroblastoma cells (SH-SY5Y cells) were provided by the Cell Culture Center of the Chinese Academy of Medical Sciences (China). This study was carried out in strict accordance with the recommendations in the Guide for the Care and Use of Laboratory Animals, 8th Edition. Primary cortical neurons and hippocampal NSCs were derived from the cerebral cortices and hippocampi of 24-hour-old Sprague Dawley rats which were purchased from the Animal Experiment Center of Southern Medical University (license no. 4402101947), and the protocols were approved by the Committee on the Ethics of the Institute of Laboratory Animal Science, Jinan University.

G-Rg1 was obtained from the National Institute for the Control of Pharmaceutical and Biological Products (China) with a molecular weight of 800, a melting point of 194-195°C, in the white powder-like crystals, the general formula C_42_H_72_O_14_, and a purity of over 98% documented by reverse-phase high-pressure liquid chromatography.* T*-BHP was purchased from Aladdin (China).

### 2.2. Cell Culture

SH-SY5Y cells were grown in DMEM supplemented with 10% FBS. Cultures were maintained in a humidified incubator at 37°C in an atmosphere of 5% CO_2_ and 95% air as described by American Type Culture Collection (ATCC) protocol.

The protocols of primary cortical neurons were on the basis of previously described [25], with a slight modification. Briefly, after anesthesia by intramuscular injection of 20% pentobarbital sodium (200 mg/mL, 0.5 mL/kg), newborn Sprague-Dawley rats were sacrificed and their brains were collected for neuron and hippocampal NSCs culture. After the hippocampi were identified as a seahorse-shaped structure in medial temporal lobes, hippocampi or cortex was, respectively, dissected out under a stereomicroscope and collected in cold sterile Hank's Buffered Salt Solution (HBSS; Life Technologies Inc., Hong Kong, China) immediately. The tissues were cut into small pieces and digested using trypsinization. Cortex cells were harvested after centrifugation at 1000 rpm, 5 minutes, and washed in High Glucose DMEM (Hyclone). Cells from cortex tissue were separately plated on polylysine-coated coverslips, in dulbecco's modified eagle's medium (DMEM)/F12 (1 : 1) supplemented with 10% FBS, and cell suspension was added, 5 × 10^5^/well, to 6-well plate. After 4 hours, the medium was replaced by neurobasal medium supplemented with 2% B27 (NCM) and exchanged with complete medium every three days. After 5 days, we experimented with the cortex neurons. The hippocampal cells were resuspended in HBSS to obtain single-cell suspensions which were plated onto a 25 cm^2^ cell culture flask at a density of 1 × 10^5^/mL in proliferation medium (DFCM; (dulbecco's modified eagle's medium (DMEM)/F12 (1 : 1), B27 supplement, 20 ng/mL basic fibroblast growth factor, and 20 ng/mL epidermal growth factor; Life Technologies Inc.)) for NSCs. The cells were incubated in an incubator at 37°C with 5% CO_2_ and supplied complete medium (primary DFCM: supply DFCM = 1 : 1) every three days [26]; meanwhile, the NSCs population (neurosphere) was separated by Pasteur Straw, purified after density centrifugation at 500 rpm, and the primary DFCM was harvested after centrifugation at 4000 rpm, and then supplied to the purified NSCs as the half of supplied complete medium. After 9 days, we experimented with the NSCs.

### 2.3. Transient Transfection of SH-SY5Y Cells and NSCs

According to the GenBank, the full-length cDNA of HSP70 was L16764.1. By analysis of Primer Premier 5.0 software, two primers of HSP70 were determined as follows: forward HSP70 (5′CGG-AAT-TCG-ATG-GCC-AAG-AAA-ACA-GCG-ATC-G-3′) and reverse HSP70 (5′CGC-GGA-TCC-GCG-TAA-TCC-ACC-TCC-TCG-ATG-G-3′). Forward primer induced the enzyme loci of* Eco R* I; reverse primer induced the the enzyme loci of* Bam H* I.

Total RNA, containing miRNAs, was isolated from fetal liver tissue of Sprague-Dawley rats. The full-length cDNA of HSP70 was amplified by RT-PCR and cloned into an eukaryotic expression vector containing the enhanced green fluorescent protein (EGFP) reporter gene pEGFP-C2. Sequencing analysis was performed to conform the recombinant plasmid pEGFP-C2-HSP70 (pEGFP-HSP70) (these data which have been published in Chinese Journal of Pathophysiology).

SH-SY5Y cells were transient transfected with the pEGFP-HSP70, using Transfection Reagent FuGene HD for 24 hours. The transfection efficiency was evaluated by counting the number of EGFP-positive cells using immunofluorescence detection. Western blot was performed to detect HSP70 protein last transfected for 24 hours. The SH-SY5Y cells were separated into three groups: (1) control (nontransfected), (2) pEGFP (pEGFP-C2-transfected), and (3) pEGFP-HSP70 (pEGFP-C2-HSP70-transfected). Western blot was carried out using standard protocols. The SH-SY5Y cells were lysed on ice with PMSF lysis buffer (Applygen Technologies Inc, Beijing, China) for 30 minutes. Lysed cells were centrifugated at 12,000 ×g for 15 minutes at 4°C to obtain total protein. A total of 38 *μ*g of protein was separated by sodium dodecyl sulfate polyacrylamide gel electrophoresis on 5% to 10% gels and then transferred to polyvinylidene fluoride membranes (Merck Millipore, Billerica, MA, USA). The membranes were blocked with 5% skimmed milk powder in TBST (10 mmol/L Tris-HCl, pH 7.5, 150 mmol/L NaCl, 0.05% Tween-20) and incubated with rabbit anti-HSP70 (1 : 1000, Cell Signaling, Boston, MA, USA) and rabbit anti-*β*-actin (1 : 1,000, Cell Signaling, Boston, MA, USA) antibody at 4°C for 16 hours. After the membranes were incubated with the secondary antibody, horseradish peroxidase-labeled IgG (goat anti-rabbit IgG/HRP, 1 : 5000, Cell Signaling, Boston, MA, USA), for 1 hour and visualized with an ECL chemiluminescent reagent system (Merck Millipore, Billerica, MA, USA). The protein bands were performed with Quantity One software using *β*-actin as the control.

NSCs were transient transfected with the pEGFP-HSP70, using Transfection Reagent FuGene HD for 72 hours. The transfection efficiency was evaluated by FITC-A mean under flow cytometry. Western blot was performed to detect HSP70 protein after being transfected in Day 1, Day 3, and Day 7. The NSCs were separated into two groups: (1) control (nontransfected) and (2) pEGFP-HSP70 (pEGFP-C2-HSP70-transfection). Western blot was carried out using standard protocols. The NSCs were lysed and the next protocols were the same as HSP70 protein detected in SH-SY5Y cells.

### 2.4. Model of SH-SY5Y Cells Damage and Grouping

Effects of* t-*BHP and G-Rg1 on SH-SY5Y cells viability were evaluated using the 3-(4,5-dimethyl-2-thiazolyl)-2,5-diphenyltetrazolium bromide (MTT; Sigma Aldrich-Fluka, St. Louis, MO, USA) assay. Briefly, SH-SY5Y cells (1 × 10^4^ cells/well) were seeded into a 96-well culture plate. After adherence, the cells were treated with various concentrations of* t*-BHP (0, 125, 250, 500, 750, 1000, and 2000 *μ*mol/L) for 1 hour and a G-Rg1 (0, 5, 10, 20, 40, 60, and 80 *μ*mol/L) for 24 hours. The medium was removed, and cells were incubated with 100 *μ*L (0.5 mg/mL) of MTT for 4 hours at 37°C. The formazan crystals were solubilized with 150 *μ*L dimethyl sulfoxide (DMSO), and the 96-well plate was shaken for 10 minutes, and the optical density (OD) values were detected by a microplate reader using a detection wavelength of 570 nm (Tecan, Switzerland).

According to the MTT results, cortex neurons were randomly divided into 6 groups: (1) C′ (cells were treated with 5 mL DCM, *n* = 3), (2) T′ (cells were treated with 500 *μ*mol/L* t*-BHP which was diluted to 5 mL DCM for 1 hour, *n* = 3), (3) G (cells were treated with 500 *μ*mol/L* t-*BHP which contained 10 *μ*mol/L G-Rg1 for 1 hour, after being pretreated with 10 *μ*mol/L G-Rg1 for 4 hours, *n* = 3), (4) H′ (cells were treated with 500 *μ*mol/L* t*-BHP 1 hour after 24 hours transfection, *n* = 3), (5) P′ (cells were treated with 500 *μ*mol/L* t*-BHP which contained 10 *μ*mol/L G-Rg1 for 1 hour, after being pretreated with 10 *μ*mol/L G-Rg1 for 4 hours, *n* = 3), and (6) HG′ (cells were treated with 500 *μ*mol/L* t*-BHP which contained 10 *μ*mol/L G-Rg1 for 1 hour, after being pretransfected with pEGFP-HSP70 for 24 hours and pretreated with 10 *μ*mol/L G-Rg1 for 4 hours. *n* = 3).

### 2.5. Measurement of Reactive Oxygen Species Production in SH-SY5Y Cells

Reactive oxygen species (ROS) production in SH-SY5Y cells was measured by flow cytometric analysis (FACS). Cells were treated as description of grouping above, digested with 0.25% trypsin, and then collected in a centrifuge tube. DCFH-DA (10 mM) was diluted 1,000-fold in serum-free medium and used to resuspend the SH-SY5Y cells and then incubated for 20 minutes at 37°C. The cells were washed with PBS to remove extracellular DCFH-DA and resuspended in 200 *μ*L PBS. Cells were subjected to FACS using a FACS Calibur (BD Biosciences, San Jose, CA, USA), and 10,000 cells from each group were acquired to determine DCF fluorescence. The data were analyzed using Cell Quest software, and the DCF fluorescence of each treatment group was compared with that of C′.

### 2.6. Analysis of DNA Damage

Comet assay was performed to detect DNA damage. Cells were cultured and treated as described above, and samples were mixed with 1% low-melting point (LMP) agarose at 37°C before being spread on a glass slide precoated with 1% normal-melting point (NMP) agarose. Then the slides were permeated in a freshly cold lysis solution (2.5 mol/L NaCl 100 mmol/L EDTA, 10 mmol/L Tris, pH = 10, 1% Triton X-100 and 10% DMSO) for 1 hour in the dark and then were washed in a freshly alkaline electrophoresis buffer (0.3 mol/L NaOH, 1 mmol/L Na2EDTA, pH > 13) for 2 hours. DNA was electrophoresed at 25 V. After that, the slides were washed in a neutralizing buffer (0.4 mol/L Tris, pH 7.5) and stained with 30 *μ*g/mL ethidium bromide. Optical images were obtained with a fluorescent microscope with 40x objective (Leica, DM6000B, Germany). The damage profile of DNA was assessed by scoring the tail lengths of the comets for at least 25 cells on each slide which were randomly selected via comet assay software.

### 2.7. Immunofluorescence Detection of Cholinergic Nervous Cells Derived from NSCs

Hippocampal NSCs were purified after density centrifugation and cultured in 12-well plates for 3 days according to the following study. Samples were fixed with 4% paraformaldehyde (PFA), blocked by goat serum (diluted into PBST; 0.01 mol/L PBS, 0.02% Triton-100), and immunostained with mouse anti-Nestin (1 : 500; Merck Millipore, Billerica, MA, USA) for 24 hours in the dark at 4°C. Sections were then incubated with secondary antibodies (Dy-Light 488 AffiniPure goat anti-mouse IgG (1 : 250, Jackson, PA, USA) for 1 hour in the dark at 37°C. Nuclei were counterstained with Hoechst 33342 (Beyotime Institute of Biotechnology, China). Sections were observed under a fluorescence microscope. Pictures were captured under fluorescence microscope with 10x objective.

### 2.8. Morphological and Immunological Properties Observation of NSCs

The NSCs were separated into four groups: (1) control (NSCs treated with DFCM), (2) G-Rg1 (NSCs treated with G-Rg1 which dissolved into DFCM), (3) pEGFP-HSP70 (after being transfected with pEGFP-HSP70, NSCs were treated with DFCM), (4) pEGFP-HSP70+G-Rg1 (after being transfected with pEGFP-HSP70, NSCs were treated with G-Rg1 which dissolved into DFCM). After cells were cultured in vitro for 1, 3, and 7 days, pictures were captured under light microscope with 100x objective (Carl Zeiss, Germany). To assess the NSCs viability which were treated as grouping above at a density of 1 × 10^5^/mL, the MTT assay was performed as described by American Type Culture Collection (ATCC) protocol. According to the MTT results, immunofluorescence of ChAT was detected in Day 3 and cells were immunostained with goat anti-ChAT (1 : 500; Merck Millipore, Billerica, MA, USA). Pictures were captured under fluorescence microscope with 40x objective. Western blot was carried out using standard protocols to detect the expression of HSP70 and ChAT protein in Day 3.

### 2.9. Neuron-NSCs Coculture System Model

*T*-BHP which was diluted to 5 mL NCM to 10 *μ*mol/L and cortex neurons which were neurons culture were plated onto a 3.5 cm^2^ cell culture flask at a density of 1 × 10^5^/mL and randomly divided into seven groups: (1) C (neurons culture was treated with 5 mL NCM for 3 days, *n* = 3), (2) T (neurons culture was treated with* t*-BHP for 3 days, *n* = 3), (3) G (neurons culture was treated with* t-*BHP which contained 10 *μ*mol/L G-Rg1 for 68 hours after being pretreated with 10 *μ*mol/L G-Rg1 for 4 hours, *n* = 3), (4) N (neurons culture was directly cocultured with NSCs (at a density of 1 × 10^4^/mL), meanwhile treated with* t*-BHP for 3 days, *n* = 3), (5) NG (neurons culture was directly cocultured with NSCs (at a density of 1 × 10^4^/mL), treated with* t-*BHP which contained 10 *μ*mol/L G-Rg1 for 68 hours after being pretreated with 10 *μ*mol/L G-Rg1 for 4 hours, *n* = 3), (6) NH (neurons culture was directly cocultured with NSCs [at a density of 1 × 10^4^/mL], treated with* t*-BHP for 3 days after being pretransfected with pEGFP-HSP70 for 3 days, *n* = 3), (7) NHG (NSCs were pretransfected with pEGFP-HSP70 for 3 days and followed neurons culture which was directly cocultured with transfected NSCs (at a density of 1 × 10^4^/mL), and treated with* t*-BHP which contained 10 *μ*mol/L G-Rg1 for 3 days after neurons were pretreated with 10 *μ*mol/L G-Rg1 for 4 hours, *n* = 3).

### 2.10. Neurites Analysis by 3D Reconstruction

Cortex neurons were seeded in 12-well plates and treated as coculture protocols. After that, neurons were fixed with 4% PFA and immunostained with mouse anti-rat microtubule-associated protein 2 (MAP-2; 1 : 500; Cell Signaling, Boston, MA, USA). Pictures were captured under fluorescence microscope, and the lengths and number of neurites (filaments) were analyzed using the Imaris software (BitPlane AG) [10].

### 2.11. Morphological and Immunological Properties of Coculture Nervous Cells

The NSCs were separated into seven groups as the coculture protocols. The samples were rinsed with PBS and fixed with 2.5% glutaraldehyde (diluted in 0.1 mmol/L PBS, PH 7.2~7.4). After that, the sections were dehydrated with the different concentration ethanol (70%, 80%, 90%, 100%, and 100% for every five minutes) and displaced by isoamyl acetate. Pictures were captured under scanning electron microscope (SEM; Carl Zeiss, Germany) after conventional critical point drying and gold-plating.

Nervous cells in coculture system (NSCs, neurons and glia) were, respectively, cultured in 12-well plates as the coculture protocols. Samples were fixed with 4% PFA, blocked by goat serum which was diluted into PBST, and immunostained with rabbit anti-HSP70 (1 : 100, Cell Signaling, Boston, MA, USA) and mouse anti-synaptophysin (1 : 500; Millipore, MA, USA) for 24 hours in the dark at 4°C. Sections were then followed by incubation with secondary antibodies (Fluorescein (FITC) AffiniPure goat anti-rabbit IgG (1 : 250, Jackson, PA, USA); DyLight 594 AffiniPure goat anti-mouse IgG (1 : 250; Jackson, PA, USA)). Pictures were captured under fluorescence microscope with 40x objective.

### 2.12. Western Blot Analysis

Cells in coculture system were plated in 6-well plates. Western blot was performed to detect the following protein expression. Samples were incubated with rabbit anti-HSP70 (1 : 1000, Cell Signaling, Boston, MA, USA), Goat anti-ChAT (1 : 800; Merck Millipore, Billerica, MA, USA), mouse anti-NeuN (1 : 1000; Merck Millipore, Billerica, MA, USA), mouse anti-GFAP (1 : 1000; Merck Millipore, Billerica, MA, USA), mouse anti-synaptophysin (1 : 1000; Millipore, MA, USA), rabbit anti-cleaved-Caspase-3 (1 : 1000, Cell Signaling, Boston, MA, USA), and rabbit anti-*β*-actin (1 : 1000; CST Inc. Danvers, MA, USA) antibody for 16 hours at 4°C. After incubating with the secondary antibody, horseradish peroxidase-labeled IgG was visualized with an ECL chemiluminescent reagent system. The protein bands were performed with Quanti Scan software using *β*-actin as control which had been standardized to 1.

### 2.13. Statistical Analysis

The data were analyzed using SPSS 13.0 statistical software. In cases of multiple comparisons, data were analyzed using a one-way analysis of variance (one-way ANOVA). In cases of repeated multiple comparisons, data were analyzed using Univariate Analysis. Values are presented as mean ± SEM. Student's *t*-test was performed for statistical evaluation. Differences with a level of *P* < 0.05 were considered statistically significant.

## 3. Results

### 3.1. Functional Properties of HSP70 and G-Rg1

**Figure 1:**
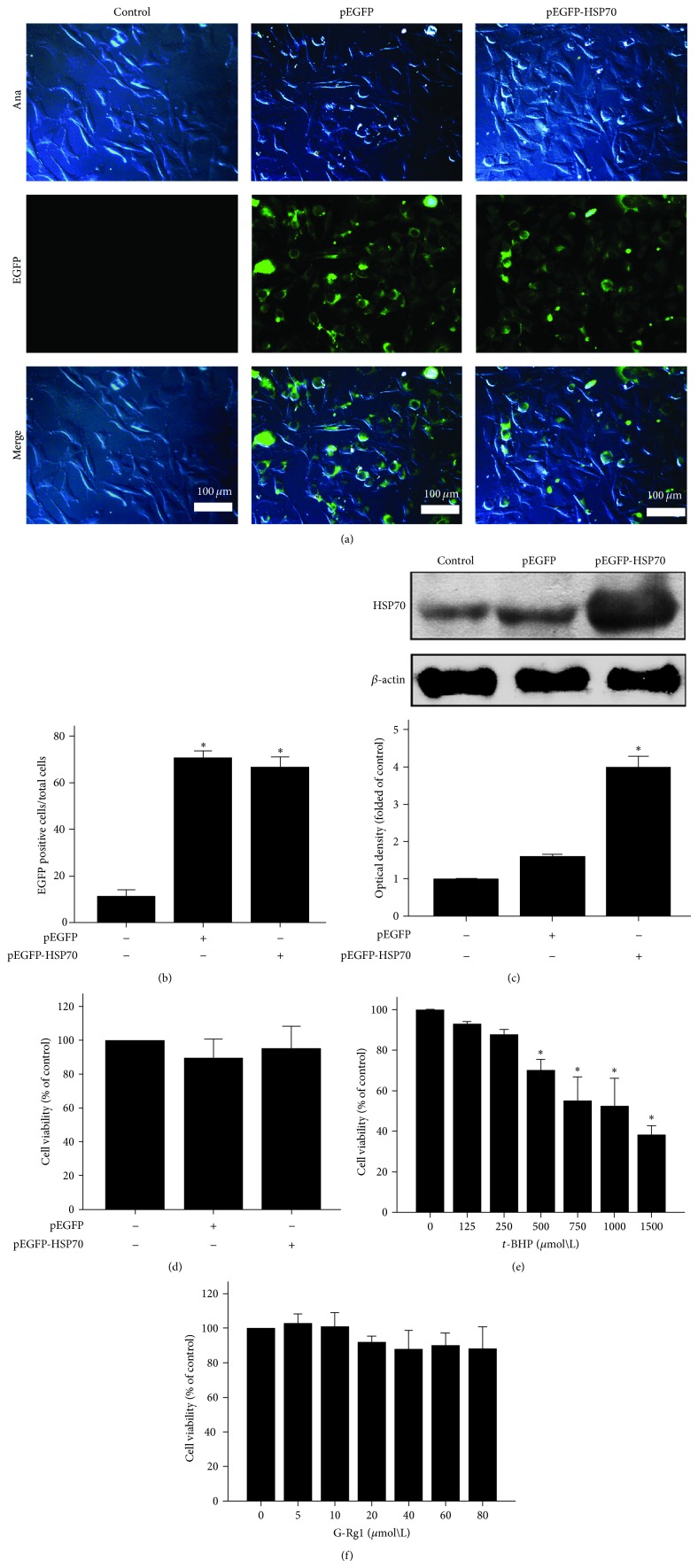
Effects of pEGFP-HSP70, t-BHP, and G-Rg1 on SH-SY5Y cell viability. (a) The expression of HSP70 secreted by SH-SY5Y was captured by light microscope in C (media control), pEGFP (transfected with pEGFP in media), and pEGFP-HSP70 (transfected with pEGFP-HSP70 in media) groups. (b) Cells were subjected to the MTT assay to detect whether our recombination plasmid pEGFP-C2-HSP70 affected cell viability in pEGFP-HSP70 compared to C. Statistical comparisons were carried out with Student's *t*-test. Values were presented as mean ± SEM. *n* = 3. (c) HSP70 protein was detected by Western Blot analysis. Statistical comparisons were carried out with Student's *t*-test. Values were presented as mean ± SEM. ^*^ *P* < 0.05; experimental group versus medium control. *n* = 3. (d) Compared to media control group, cells after being transfected were used to the MTT assay for viability analysis, respectively. Statistical comparisons were carried out with Student's *t*-test. Values were presented as mean ± SEM. *n* = 3. The two groups which were transfected with pEGFP and pEGFP-HSP70 versus medium control. (e) SH-SY5Y cells were treated with* t*-BHP at various concentrations (0, 125, 250, 500, 750, 1000, and 1500 *μ*mol/L) for 1 hour. (f) G-Rg1 at various concentrations for 24 hours. Cells were subjected to the MTT assay for viability analysis, respectively. Statistical comparisons were carried out with one ANOVA. Values were presented as mean ± SEM. ^*^ *P* < 0.05 versus medium group. *n* = 3.

To overexpress the HSP70 gene, SH-SY5Y cells were exposed to the produced plasmid encoding the rat HSP70-EGFP transgene. EGFP served as the control by marking all cells that received the target plasmid carrying the HSP70 gene. 67% FITC-A under fluorescent microscopy was increased for 24 hours (Figures 1(a) and 1(b)). Western blot was performed to assess the HSP70 protein expression which has increased by about 250%, compared to media control and pEGFP control (Figure 1(c)); meanwhile, MTT assay was performed to demonstrate that the plasmid did not affect the SH-SY5Y cell viability compared to the media control which has neither Transfection Reagent FuGene HD nor plasmid (Figure 1(d)).

We used the MTT assay to study the effects of* t*-BHP and G-Rg1 on SH-SY5Y cells viability.* T*-BHP reduced SH-SY5Y cell viability in a dose-dependent manner. Compared with medium control (100% of SH-SY5Y cell viability), SH-SY5Y cell viability was significantly reduced to 70%, 55%, 52%, and 38% in the doses at 500, 750, 1000, and 1500 *μ*mol/L, respectively (Figure 1(e)). We chose* t*-BHP at 500 *μ*mol/L which is the lowest dose in inducing a significant reduction of SH-SY5Y cell viability in 1 hour. The MTT assay was also employed to determine the effect of G-Rg1 which exhibits any overtly toxic effects on SH-SY5Y cells, whereas G-Rg1 at 20 *μ*mol/L reduced 8% SH-SY5Y cell viability in baseline level without statistical differences (*P* > 0.05) (Figure 1(f)). Thus, G-Rg1 at 10 *μ*mol/L was chosen as the experimental concentration in the studies of anti-*t*-BHP neurotoxicity.

**Figure 2:**
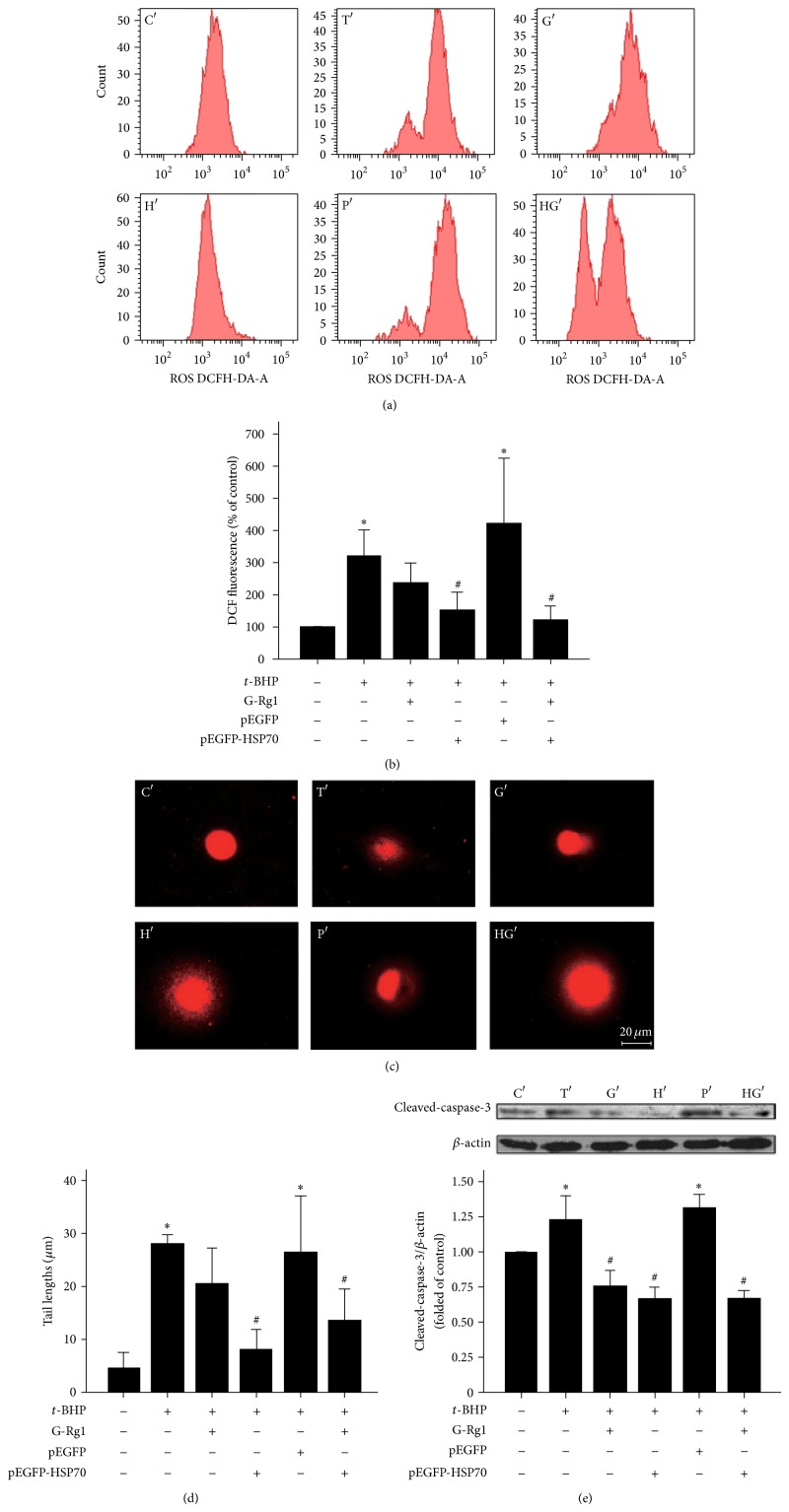
Functional properties of HSP70 and G-Rg1 on t-BHP-induced SHSY5Y cell injury. (a) ROS production in SHSY-5Y was determined using BD FACS. (b) ROS production was shown by DCF fluorescence in C′ (media control), T′ (10 *μ*mol/L* t*-BHP), G′ (10 *μ*mol/L G-Rg1 and 10 *μ*mol/L* t*-BHP), H′ (SHSY-5Y cultured in 10 *μ*mol/L* t*-BHP after being transfected with pEGFP-HSP70), P′ (SHSY-5Y cultured in 10 *μ*mol/L* t*-BHP after being transfected with pEGFP), and HG′ (SH-SY5Y cultured in 10 *μ*mol/L G-Rg1 and 10 *μ*mol/L* t-*BHP after being transfected with pEGFP-HSP70), respectively. Statistical comparisons were carried out with Student's *t*-test. Values were presented as mean ± SEM. *n* = 3. T′, G′, H′, P′, and HG′ versus C′; ^#^ *P* < 0.05 versus T′. (c) DNA fragment in SH-SY5Y cells was determined using fluorescence microscope. (d) DNA fragment was described by tail lengths which were assessed by CAMP software in C′, T′, G′, H′, P′, and HG′, respectively. Statistical comparisons were carried out with Student's *t*-test. Values were presented as mean ± SEM. *n* = 3. T′, G′, H′, P′, and HG′ versus C′; ^#^ *P* < 0.05 versus T′. (e) SH-SY5Y cells were then subjected to apoptosis analysis. Apoptosis of neurons was determined by Western blot analysis of cleaved Caspase-3 levels. Statistical comparisons were carried out with Student's *t*-test. Values were presented as mean ± SEM. ^*^ *P* < 0.05 versus C′; ^#^ *P* < 0.05 versus T′. *n* = 3.

To investigate whether G-Rg1 and overexpression HSP70 reduce the production of ROS, we used flow cytometry to measure ROS levels in C′ group, T′ group, G′ group, H′ group, P′ group, and HG′ group. We found that ROS levels in SH-SY5Y cells treated with* t*-BHP were increased about 221% compared to C′ group; in contrast, ROS level in G′, H′, and HG′ groups was decreased by 136%, 74%, and 126%, respectively, compared to C′ group. Statistical analysis showed that the production of ROS was significantly decreased in H′ and HG′ group (*P* < 0.05), whereas G-Rg1 (10 *μ*mol/L) combined with overexpression of HSP70 did not significantly inhibit the production of ROS than G′ group and H′ group (*P* > 0.05) (Figures 2(a) and 2(b)).

ROS is known to diffuse into the neuron nucleus and pose serious damage in DNA. Theoretically, apoptotic cells with extensive DNA internucleosomal fragments can be detected by this method [27]. Comet assay used alkaline single cell gel electrophoresis to detect DNA fragments in the cells, and then apoptotic cells showed a comet feature with a very small head and a large and wide tail. The mean tail moments increased at 1 hour but were significantly decreased in H′ group and HG′ group compared to C′ group (*P* < 0.05) (Figures 2(c) and 2(d)).

Next, we investigated whether overexpression of HSP70 and G-Rg1 affect* t*-BHP-induced cell apoptosis through the Caspase-dependent pathway. Compared with C′ group, Caspase-3 activation (cleaved-Caspase-3) was significantly increased in T′ group (*P* < 0.05), whereas it is decreased in G′, H′, and HG′ groups, respectively. The effects by each of these reagents were also significant (*P* < 0.05) (Figure 2(e)).

The results of cotreatment were not significantly different among G′ group, H′ group, and HG′ group (*P* > 0.05) because of the properties of the cell lines and the short time points of damage. Clonal analysis in the adult mouse hippocampus had demonstrated that hippocampal NSCs were multipotent and could generate both neurons and astrocytes and that they used two modes of divisions to self-renew [28]. We chose hippocampal NSCs as the vehicle of HSP70 gene into the neuron growth microenvironment since NSCs were the resident cells which were at rest in normal brain and were proliferated and differentiated in the lesions of the chronic disease brain. In AD brain, the forebrain cholinergic nervous cells loss is more terrible than that of other parts, whereas the NSCs from hippocampus which could migrate to the forebrain are the best source cells. So the supplement of NSCs from hippocampus to treat AD should be currently one of the hottest research fields in biology [29]. In the present study, we took neurons-NSCs coculture system to imitate the neuron damage microenvironment [30].

### 3.2. Morphological and Immunological Properties of NSCs in Different Doses and Transfected NSCs from Hippocampus

**Figure 3:**
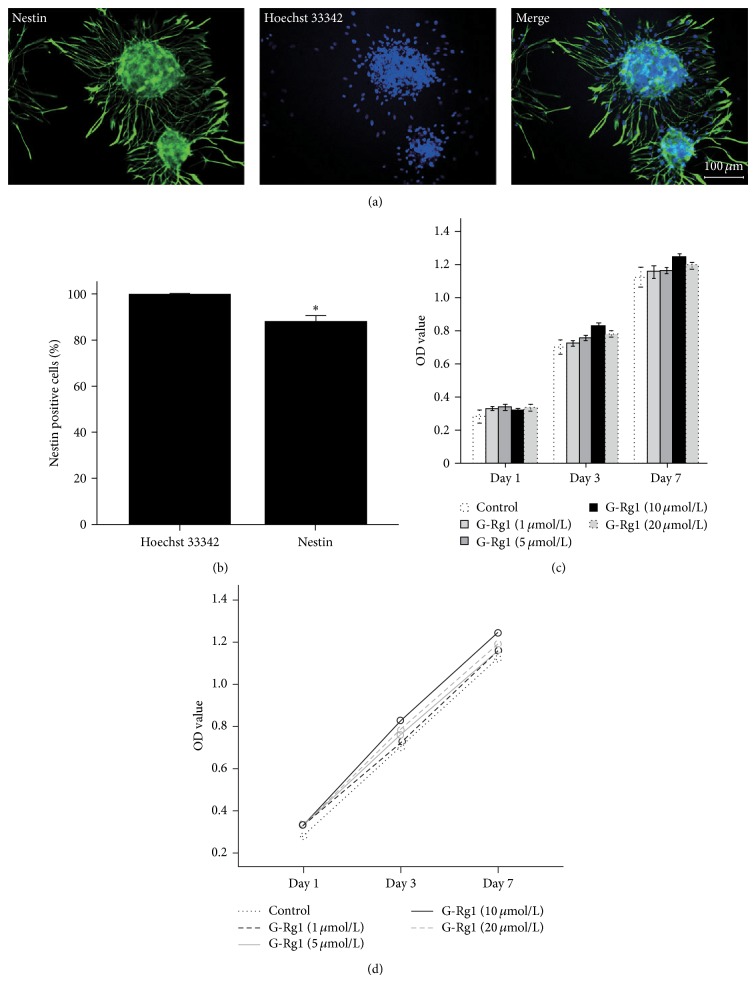
G-Rg1 stimulated NSCs proliferation. (a) Immunofluorescence analysis of hippocampal NSCs cultures treated with media in 3rd day, stained with antibodies against the NSC-specific Nestin (green), and nuclei were counterstained with Hoechst 33342 (blue). (b) The nestin positive cells were counted by Image J software; statistical comparisons were carried out with Independent *t*-test. Values were represented as mean ± SEM. Nestin positive cells versus Hoechst-stained cells. *n* = 9. (c) The MTT assay indirectly showed the OD value of NSCs proliferation for 7 days after being treated with different concentration of G-Rg1. Statistical comparisons of repeated multiple comparisons were carried out with Univariate Analysis; Statistical comparisons of multiple comparisons in the group of each time point were, respectively, carried out with Student's *t*-test. Values were represented as mean ± SEM. *n* = 3. (d) The MTT assay was described by Profile Plots.

In the current experiment, we performed an immunofluorescence analysis to examine the expression of stem cells markers Nestin in NSCs, whose results showed that Nestin positive cells were 86% in total cells in Day 3 (Figure 3(a)). A growth effects of G-Rg1 (1, 5, 10, and 20 *μ*mol/L) on NSCs were examined by MTT assay following 1, 3, and 7 days of culture (Figures 3(b) and 3(c)), while the cell viability in 10 *μ*mol/L G-Rg1 group was higher than other cells viability in 1, 5, and 20 *μ*mol/L groups in Day 3 and Day 7.

**Figure 4:**
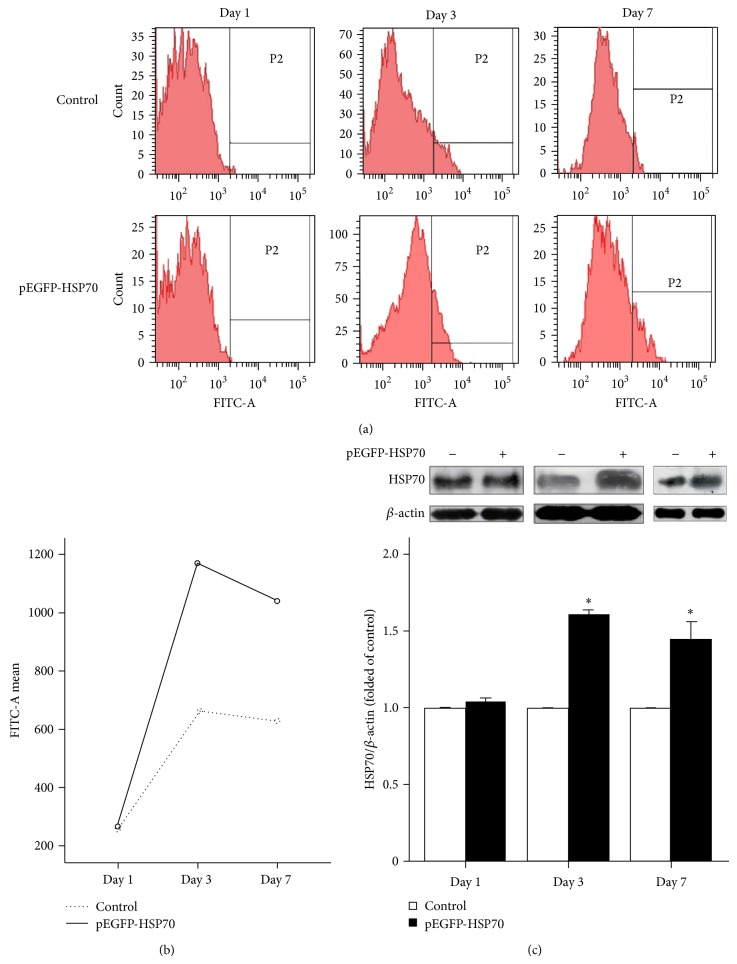
Efficiency of transfection of NSCs used pEGFP-C2-HSP70 vector. (a) Efficiency of transfection was confirmed by visualizing EGFP expression after transfection in 1, 3, and 7 days. Flow cytometry was performed to detect the fluorescence intensity of EGFP in Day 1, Day 3, and Day 7. (b) Fluorescence intensity of EGFP assessed by flow cytometry was described by Profile Plots. (c) Western Blot was performed to detect HSP70 protein after transfection in Day 1, Day 3, and Day 7. Statistical comparisons were carried out with Independent *t*-test. Values were presented as mean ± SEM. ^*^ *P* < 0.05 versus control in Day 3; ^*^ *P* < 0.05 versus control in Day 7.

To overexpress the HSP70 gene, NSCs as vehicle for HSP70 gene transfer were exposed to the produced plasmid encoding the rat HSP70-EGFP transgene, and observation of EGFP positive cells by flow cytometry showed that almost 66% FITC-A was increased from Day 3 (Figures 4(a) and 4(b)). Western blot was performed to detect the HSP70 protein of nontransfected and transfected NSCs in Day 1, Day 3, and Day 7. The obtained results showed that NSCs secreted detectable amounts of HSP70 which was relatively highest in Day 3 (*P* < 0.05) (Figure 4(c)).

**Figure 5:**
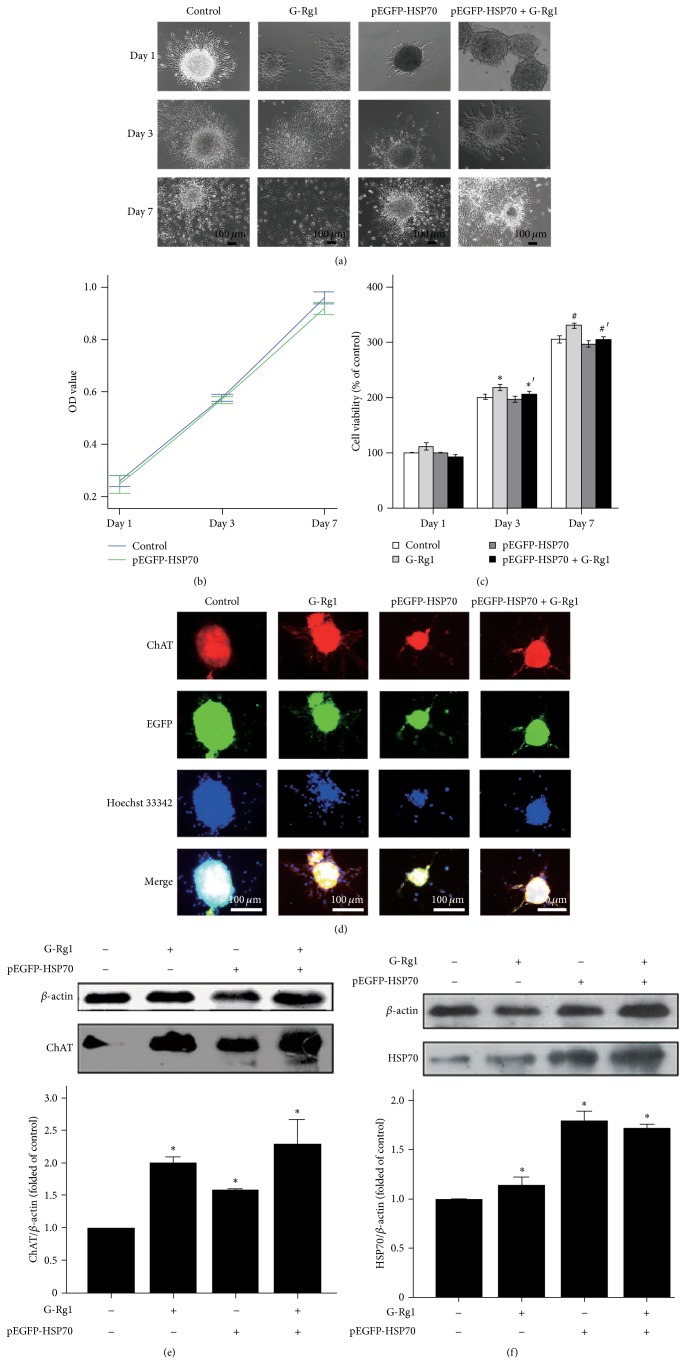
Properties of NSCs after being transfected with pEGFP-HSP70 or/and treated with G-Rg1. (a) Representative images were shown at 100x magnification to show some morphological changes of NSCs after being transfected with pEGFP-HSP70 or/and treated with G-Rg1 in Day 1, Day 3, and Day 7, such as neurospheres and branches. (b) The MTT assay showing the OD value of NSCs after being transfected with p EGFP-HSP70 in Day 1, Day 3, and Day 7. Cell viability was not significantly different between control and pEGFP-HSP70 groups at *P* > 0.05. Statistical comparisons were carried out with Independent *t*-test. Values were represented as mean ± SEM. *n* = 3. (c) The MTT assay showed the viability value of NSCs in Day 1, Day 3, and Day 7 after being transfected with p EGFP-HSP70 or/and treated with G-Rg1. ^*^ *P* < 0.05 versus media control in Day 3. ^∗′^ *P* < 0.05 versus G-Rg1 in Day 3. ^#^ *P* < 0.05 versus media control in Day 7. ^#′^ *P* < 0.05 versus control in Day 7. Statistical comparisons of repeated multiple comparisons were carried out with Student's *t*-test. Mean ± SEM. *n* = 3. (d) Immunostaining with antibodies against the cholinergic neuron-specific ChAT (red), nuclei was counterstained with Hoechst 33342 (blue) and EGFP fluorescence was green. (e) And (f) Western Blot was, respectively, performed to detect HSP70 and ChAT expression in control, G-Rg1, pEGFP-HSP70 or/and treated with G-Rg1 groups in Day 1, Day 3, and Day 7. Statistical comparisons were carried out with Student's *t*-test. Values were presented as mean ± SEM. ^*^ *P* < 0.05 versus C. *n* = 3.

After pEGFP-HSP70 transfection, morphological properties of transfected NSCs from hippocampus were observed by light microscope (Ziess, Germany) (Figure 5(a)), and an MTT assay was performed to investigate the cell viability of NSCs from hippocampus. The data indicated that transfected cells were viable morphological changes after transfection, such as the neurospheres which were less than nontransfected cells. However, there was no apparent adverse effect on cell viability and proliferation when compared to nontransfected control cells at *P* > 0.05 (Figures 5(b) and 5(c)). According to the results of the direct coculture cell state in preexperiment, we performed an immunofluorescence to manifest the expression of cholinergic nervous cells markers ChAT in NSCs (Figure 5(d)) and western blot assay to detect the level of HSP70 protein and ChAT protein in Day 3 (Figures 5(e) and 5(f)). The data showed that the ChAT expression treated with G-Rg1 medium was higher than that treated with medium; HSP70 level transfected with pEGFP-HSP70 was higher than nontransfected groups (*P* < 0.05).

### 3.3. Comparison of Neuron Injury in Different Days

**Figure 6:**
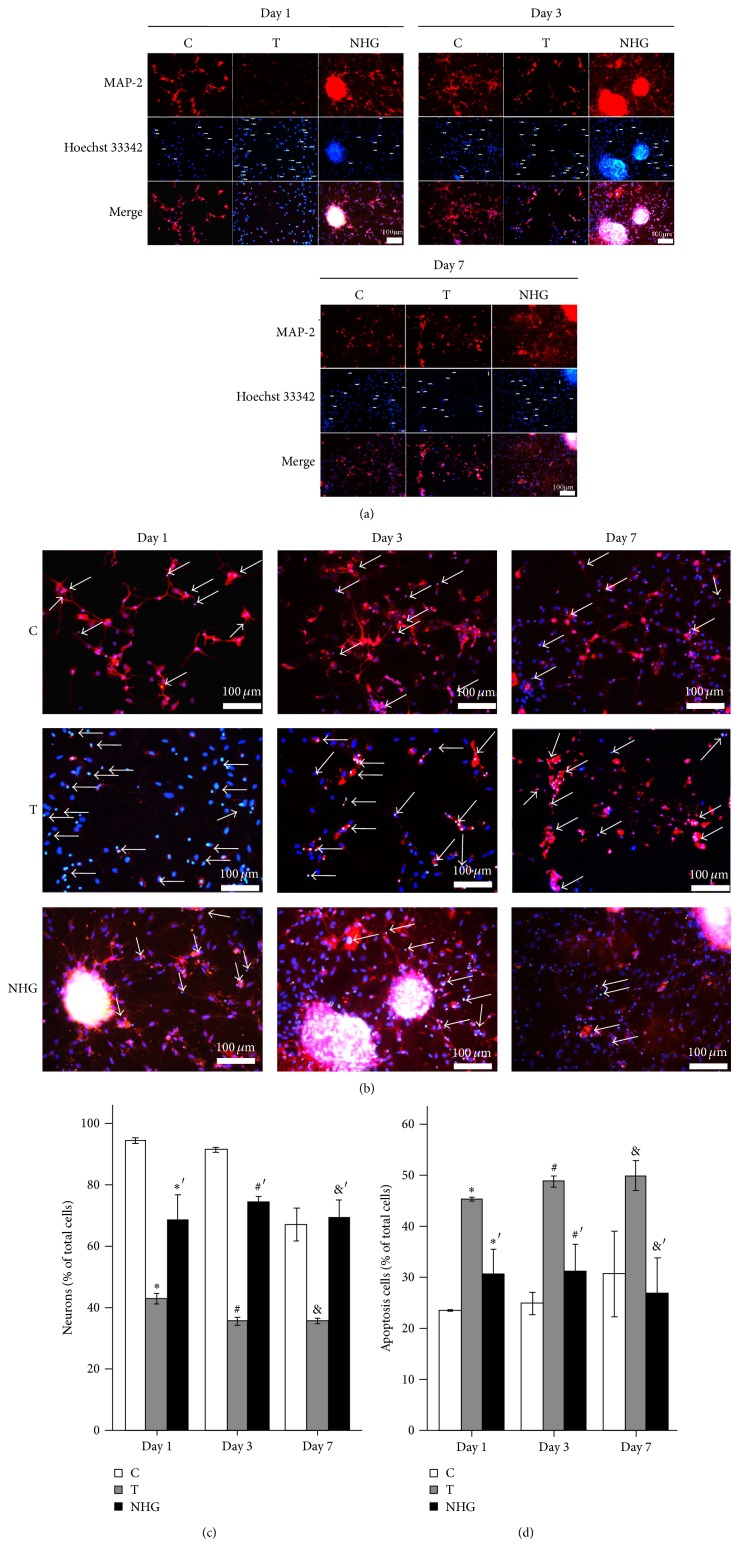
Neuronal cell count and cell apoptosis. (a) Representative images of immunofluorescence were shown at 200x magnification to demonstrate the MAP-2 positive neurons in cotreatment on* t*-BHP-induced injury. TRITC (red)-labeled MAP-2 immunoreactions were shown in the cytoplasm; Nuclei was stained with Hoechst 33342 (blue) which as a specific marker of apoptosis was applied to determine chromatin condensation. Cells with intensely stained, condensed, or fragmented nuclei were scored as apoptotic. The arrowheads indicate remarkable apoptotic cells. C: media control; T: 10 *μ*mol/L* t*-BHP; NHG: cocultured with NSCs which were transfected with pEGFP-HSP70 and G-Rg1 which dissolved in 10 *μ*mol/L* t*-BHP. (b) The merged images of (a) pictures were magnified which were convenient for observation. (c), (d) Neuronal cell count and the percentage of apoptotic neurons were scored in Day 1, Day 3, and Day 7 for ascertaining the time points of study. Statistical comparisons of repeated multiple comparisons were carried out with Student's *t*-test; statistical comparisons of multiple comparisons in the group of each time point were, respectively, carried out with Student's *t*-test. Mean ± SEM. ^*^ *P* < 0.05 versus C in Day 1, ^#^ *P* < 0.05 versus C in Day 3, ^&^ *P* < 0.05 versus C in Day 7, ^∗′^ *P* < 0.05 versus T in Day 1, ^#′^ *P* < 0.05 versus T in Day 3, and ^&′^ *P* < 0.05 versus T in Day 7. *n* = 9.

For screening the best time point, we compared the coculture cells in terms of MAP-2 expression and apoptosis cells.  Figure 6(a), B showed a 100x magnification microscopic view of cells grown in C, T, and NHG groups for 1, 3, and 7 DIV.  Figure 6(c) demonstrated that the proportion MAP-2 positive cells of total cells were 94.5 ± 1%, 91.4 ± 0.8%, and 67.1 ± 6.6% in C group in 1, 3, and 7 DIV and 42.9 ± 2%, 37.2 ± 4.3%, and 35.5 ± 0.9% in T group, while they were 68.6 ± 10%, 74.5 ± 2%, and 69.6 ± 6.8% in NHG group, so the difference of neurons among the groups between T and NHG groups was about 25.7%, 37.3%, and 34.1% in 1, 3, and 7 DIV. Slightly, there seemed to be a decrease of neuron density in a time-dependent manner after insult, while the MAP-2 positive neuron density improved by the cotreatment was much more in 3 DIV than 1 and 7 DIV.  Figure 6(d) demonstrated that the apoptotic neurons of total cells were 45.27 ± 0.47%, 48.80 ± 1.3%, and 49.96 ± 3.6% in T group in 1, 3, and 7 DIV, while they were 30.76 ± 5.8%, 31.24 ± 6.5%, and 26.94 ± 8.4% in NHG group, so the survival cells were, respectively, increased about 14.15%, 17.56%, and 23.2% in 1, 3, and 7 DIV. Apparently, cell apoptosis was significantly inhibited after cotreatment, compared to T group (*n* = 9, ANOVA, *P* < 0.05). We comprehensively selected the groups in Day 3 to be studied, according to both the increase survival neurons and the purity of primary neurons in different days above.

### 3.4. Cotreatment Decreased Cells Apoptosis in Coculture System

**Figure 7:**
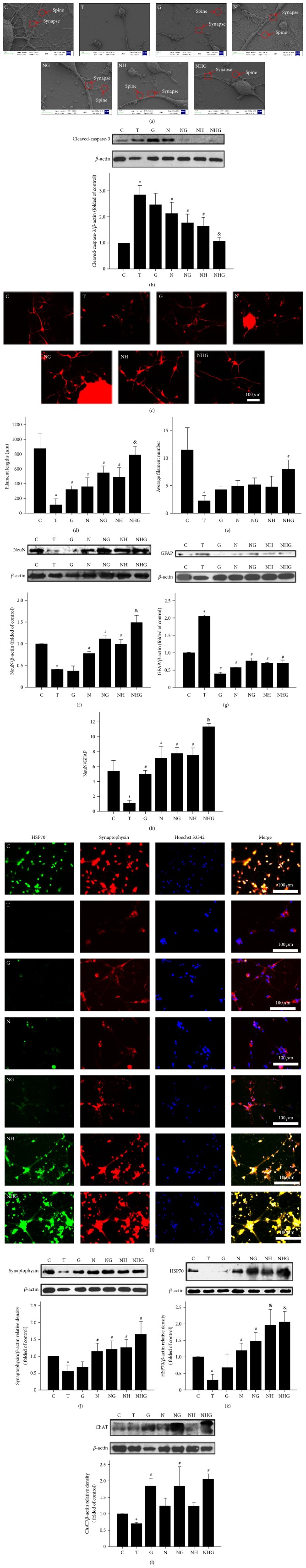
Effects of cotreatment on the damage cortex neurons. Neurons were grouped as C: media control; T: 10 *μ*mol/L* t*-BHP; G: treated with G-Rg1 which dissolved into 10 *μ*mol/L* t*-BHP; N: cocultured with NSCs in 10 *μ*mol/L* t*-BHP; NG: cocultured with NSCs and treated with G-Rg1 which dissolved into 10 *μ*mol/L* t*-BHP; NH: cocultured with overexpression of HSP70 NSCs in 10 *μ*mol/L* t*-BHP NHG: cocultured with overexpression of HSP70 NSCs and G-Rg1 which dissolved in 10 *μ*mol/L* t*-BHP. (a) Morphological changes of neurons, such as spines, filaments, and synapsis were pictured under SEM in C, T, G, N, NG, NH, and NHG at Day 3. (b) Western blot was performed to detect cleaved-Caspase-3 expression. Statistical comparisons were carried out with Student's *t*-test. Mean ± SEM. ^*^ *P* < 0.05 versus C group, ^#^ *P* < 0.05 versus T group, and ^&^ *P* < 0.05 versus monotherapy group. *n* = 3. (c) Representative images of immunofluorescence were shown at 200x magnification to demonstrate the MAP-2 positive cells; neurite lengths and number of neurites of each neuron were measured by Imaris software. (d), (e) The value of neurite lengths and number of neuritis were expressed as the mean ± SEM. Statistical comparisons were carried out with Student's *t*-test. ^*^ *P* < 0.05 versus C group, ^#^ *P* < 0.05 versus C group, and ^&^ *P* < 0.05 versus monotherapy group. *n* = 25. (f) Western blot was performed to detect NeuN and GFAP expression. (g), (h) The ratios of NeuN/*β*-actin and GFAP/*β*-actin were compared with C which had been standardized to 1. Statistical comparisons were carried out with Student's *t*-test. Values were presented as mean ± SEM. ^*^ *P* < 0.05 versus C group, ^#^ *P* < 0.05 versus T group, and ^&^ *P* < 0.05 versus monotherapy group (T, G, N, NG, and NH). *n* = 3. (i) Representative images of immunofluorescence were shown at 400x magnification to demonstrate the synaptic connection which were marked with antibody synaptophysin. (j), (k), and (l) Western blot was performed to detect HSP70, synaptophysin, and ChAT expression. Statistical comparisons were carried out with Student's *t*-test. Mean ± SEM. ^*^ *P* < 0.05 versus C group, ^#^ *P* < 0.05 versus T group, and ^&^ *P* < 0.05 versus monotherapy group. *n* = 3.

We investigated whether overexpression of HSP70 in NSCs and G-Rg1 affect* t*-BHP-induced cell apoptosis in coculture cells. It was absolutely shown that morphological changes were observed by SEM (Figure 7(a)). Normal neurons showed short neck and neckless spines and dense network, while neurons damaged by* t*-BHP showed the outer surface of edematous flat and invaginated spine, even decrease or disappearance of spine and a large decrease or disappearance of nervous cells and network. Compared with C group, Caspase-3 activation was significantly increased in T group (*P* < 0.05) and was decreased in G, NG, NH, and NHG groups, respectively; however, cleaved-Caspase-3 expression was suppressed in NHG group higher than in monotherapy groups (Figure 7(b)).

### 3.5. Overexpression of HSP70 in NSCs and G-Rg1 Increased Filaments Regrowth and the Expression of Synaptophysin of Neurons in Coculture System

Overexpression of HSP70 in NSCs and G-Rg1 improved the spine and network better than the other monotherapy from the perspective of morphological changes. To investigate neurogenesis, we performed immunofluorescence for MAP-2 in the coculture cells system to assess filament regeneration (200x; Figure 7(c)) in Day 3, and, without doubt, the length and number of neurons in NHG group acquired the best regeneration (*P* < 0.05), the total filament lengths analyzed by the Imaris software were 1163.49 ± 55.1 *μ*m, 121.3 ± 33.6 *μ*m, 338.1 ± 17.9 *μ*m, 332.5 ± 32.9 *μ*m, 509.6 ± 88.6 *μ*m, 637.9 ± 88.4 *μ*m, and 984.3 ± 77.8 *μ*m, and cotreatment apparently improved the filament outgrowth than monotherapy (*n* = 25, ANOVA, *P* < 0.05) (Figures 7(d) and 7(e)). NeuN/GFAP represented the ratio of differentiated cells from NSCs between neurons and astrocytes; the ratio was reduced in T group, but was recovered in G, N, NG, and NH groups, and was significantly better in NHG group (*P* < 0.05) (Figures 7(f), 7(g), and 7(h)). Then the results of immunofluorescence (Figure 7(i)) and western blot are shown to demonstrate the level of HSP70 protein, ChAT, and synaptophysin expression which assess the development of the terminal junction of filament synapse. The results manifested that ChAT NSCs were markedly improved in the group G-Rg1 treated than G-Rg1 untreated-group after insult (*P* < 0.05) and synaptophysin expression was apparently increased in NHG group (*P* < 0.05) (Figures 7(j), 7(k), and 7(l)).

## 4. Discussion

Recently, neural stem cell therapy was proposed as an attractive option to investigate and treat AD [31]. However, in Lishu Duan's study, basal forebrain cholinergic neurons derived from stem cell in AD patients were more susceptible to cell death [32], and progressive neuronal loss in hippocampus was also one of the characteristics which should be reversed in AD therapy [33]. Therefore, enhancement of neurons damage resistance derived from NSCs should be prior to solve in NSCs transplantation therapy. An ex vivo gene therapy strategy of overexpressing HSP70 in hippocampal NSCs was proposed. It is reasonable to expect that genes encoding HSP70 exhibit great reparative potential to accelerate cell survival [34], so firstly, we tested and verified the effect of the recombinant plasmid pEGFP-HSP70 from our institute by SH-SY5Y cells.

Upon exposure to stressors* t*-BHP, the neuron line SH-SY5Y cells and neurons were rapidly converted to cell damage including cell apoptosis [35] and oxidative stress-related genes such as the HSP70 which were to a lesser extent [36]. Our results also suggested that overexpression HSP70 in SH-SY5Y was concerned as a trigger to initiate the protection cascade. In addition, for the following study, we had to investigate the effect of G-Rg1 that possesses both neuron protecting and neurogenesis promoting effects which has been demonstrated in the study of Wu et al. [37, 38]. In accordance with these results, they suggested that overexpression of HSP70 in SH-SY5Y or G-Rg1 could reduce SH-SY5Y cell damage by decreasing the production of ROS, but the neuroprotective effect of overexpression of HSP70 in SH-SY5Y combined with G-Rg1 did not significantly reduce SH-SY5Y cell damage than monotherapy. In fact, whether there was the synergistic effect of overexpression of HSP70 and G-Rg1 should be further educated from other aspects that we had not involved.

HSP70 provides resistance in AD since it can conformationally repair the folded and unfolded forms of protein [39], respond to A*β* toxicity [40], more specifically, resist ROS which is directly generated by A*β* oligomers, and in turn exert neurotoxic effects by displaying A*β* toxicity in the brain, but the expression of HSP70 was transitory [41]. Our present study have provided that HSP70 overexpressed NSCs significantly maintain HSP70 expression for at least 7 days in vitro (Figure 4). Interestingly, NSC is one of the best mediated vectors of gene therapy integrated into central neural system and can generate neural cell properties such as neurons and glial cells [42]. NSCs from hippocampus as the donors of cell replacement therapies could be made feasible by implementing approaches applied for neurogenesis [43]. In this study, we used G-Rg1 since its function might modulate cholinergic levels and directional differentiation into neurons. In triple staining, using different doses of G-Rg1, NSCs were led in upregulation of the expression of cholinergic neuronal markers ChAT in 10 *μ*mol/L G-Rg1. After transfection in Day 1, Day 3, and Day 7, the results suggested that cell viability was increased in medium with 10 *μ*mol/L G-Rg1 than in that without G-Rg1 from the 3rd day, while transfection had no effect on cell survival but on the scope of neurospheres which are the reasons why we had not studied in the paper. Therefore, we chose 10 *μ*mol/L G-Rg1 for 3 days to study the differentiation and cell state of NSCs after adherence.

Opposite to the putative protective role of HSP70 transitory appearance, there is progressive evidence showing that after exposure to* t*-BHP for 3 days, MAP-2 is a dendrite-specific protein that plays an important role in the development, formation, and regeneration of the nervous system [44]. We investigated the rate of cell differentiation of transfected NSCs into neurons based on their expression of MAP-2. Compared to* t*-BHP group, the number of MAP-2 positive neurons in NSCs overexpressing HSP70 plus G-Rg1 group was remarkably higher in Day 3 than in Day 1 and Day 7. This result was consistent with the findings of the study time of NSCs differentiation after adherence. However, MAP-2 did not demonstrate that NSCs have undergone neurogenesis; synaptophysin is a synaptic protein which is used as a marker of synapse formation [44]. We observed that synaptophysin expression was substantially increased in NSCs-related treatment groups (N, NG, NH, and NHG groups). ChAT is the key synthetic enzyme for acetylcholine and the important symbol of the functional activity of the cholinergic system and cholinergic nervous cells, which is the marker of neurogenesis especially in AD. This almost indicated that NSCs and G-Rg1 mainly promoted synapse formation and cholinergic system function after the development of synapses and synapsis remodeling, respectively. The other best results we expected were the increase in the ratio of cholinergic neurons, and we performed western blot assay to detect the markers of neurons and astrocytes. In T groups, since the neurons were more susceptible to cell death than glia, the ratio of NeuN/GFAP was greatly decreased; while NSCs, overexpression of HSP70 in NSCs or G-Rg1, all increase the proportion of neurons in some extent in cocultures system, and the level of neurons in NHG group was apparently increased compared to monotherapy group. Nevertheless, it just suggested that the cotreatment was progressive to improve neurons and cholinergic input; as for which cells exactly input acetylcholine, it had to be further studied.

The reduction of the decrease of nervous cells might be closely related to several different mechanisms of toxicity in AD that included Caspase-3 activation [45] and oxidative stress [46]. Here, we found that combining overexpression of HSP70 in NSCs with G-Rg1 significantly blocked apoptosis and reduced neural cytotoxicity in cocultured cells, including neuron loss and synapsis damnification. Although the cause of cell death under our experimental circumstances remains to be determined, the cotreatment (combining overexpression of HSP70 in NSCs with G-Rg1) has affected not only the Caspase-3 activation but also synapsis remodeling in which therapy was better than other monotherapy in vitro.

Objectively speaking, our study needs further investigation. (1) It just provides a margin effect of cotreatment but lots of deep mechanism have to be studied, such as the main pathway and the key points of the synergistic effects of the cotreatment, which were helpful in developing new therapy. (2) We might ignore other aspects of the cotreatment, such as anti-inflammation and decreasing A*β* oligomers. (3) The study is limited in vitro and the next is focused on in vitro research. Zheng et al.'s study demonstrates that G-Rg1 could well distribute in the brain [47], and then how to promote the synergistic effects of cotreatment in vivo is worthy of study. In summary, the properties of cotreatment suggest a safe and promising cotreatment for AD therapies; it is expected to improve the study of mechanism in vitro and try to apply in AD models.

## Supplementary Material

Graphical Abstract: Hippocampal NSCs which overexpressed HSP70 and combined with G-Rg1 (cotreatment) co-cultured with nervous cells in vitro, and were analyzed the synergistic effect of the cotreatment for t-BHP-induced nercous cells damage.

## Abbreviations

NSCs: Neural stem cells
G-Rg1: Ginsenoside Rg1
*t*-BHP: Tert-butyl hydroperoxide
HSP70: Heat shock protein 70
3D: Three-dimensional/three dimensions
ANOVA: Analysis of variance
GFAP: Glial-fibrillary acidic protein
GSK-3*β*: Glycogen synthase kinase-3*β*.
