# Supplementary figures and images for: The Synergistic Effects of Heat Shock Protein 70 and Ginsenoside Rg1 against Tert-Butyl Hydroperoxide Damage Model In Vitro
Source: Oxid Med Cell Longev. 2015 Jan 15;2015:437127. doi: 10.1155/2015/437127 (PMC4312651; doi:10.1155/2015/437127)

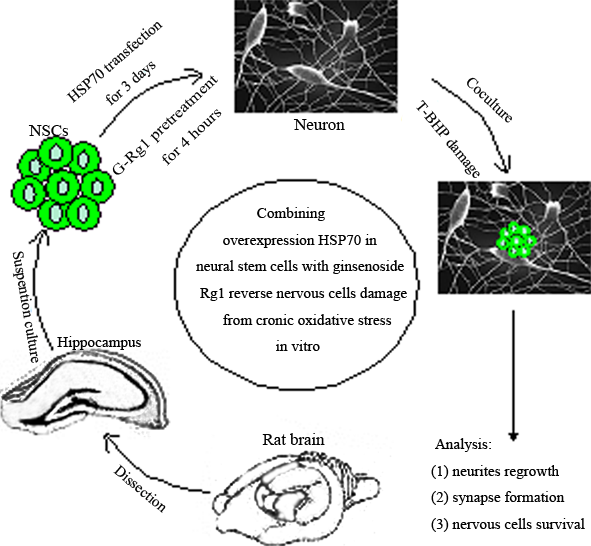

Supplement: Supplementary file 1 — Graphical Abstract: Hippocampal NSCs which overexpressed HSP70 and combined with G-Rg1 (cotreatment) co-cultured with nervous cells in vitro, and were analyzed the synergistic effect of the cotreatment for t-BHP-induced nercous cells damage. [file 437127.f1.doc]
